# Associations between breastfeeding intention, breastfeeding practices and post‐natal depression during the COVID‐19 pandemic: A multi‐country cross‐sectional study

**DOI:** 10.1111/mcn.13450

**Published:** 2022-11-09

**Authors:** Yan‐Shing Chang, Kan M. C. Li, Li‐Yin Chien, Eun Y. Lee, Seo A. Hong, Kelly P. Coca

**Affiliations:** ^1^ Florence Nightingale Faculty of Nursing, Midwifery & Palliative Care King's College London London UK; ^2^ Guy's and St. Thomas' NHS Foundation Trust Evelina London Children's Hospital London UK; ^3^ Institute of Community Health Care, National Yang Ming Chiao Tung University Yang‐Ming Campus Taipei Taiwan; ^4^ Department of Nursing Catholic Kkottongnae University Cheongju Republic of Korea; ^5^ ASEAN Institute for Health Development Mahidol University Nakhon Pathom Thailand; ^6^ Institute for Health and Society Hanyang University Seoul Republic of Korea; ^7^ Department of Women's Health Nursing, Escola Paulista de Enfermagem Universidade Federal de São Paulo São Paulo Brazil

**Keywords:** breastfeeding, breastfeeding duration, breastfeeding intention, COVID‐19, infant feeding behaviour, post‐natal depression

## Abstract

Associations between breastfeeding intention, duration and post‐natal depression (PND) have been shown in pre‐COVID‐19 studies. However, studies during COVID‐19 have not examined the associations between breastfeeding intention, breastfeeding practices, and PND in an international sample of post‐natal women, taking into consideration COVID‐19 related factors. This is the first study to address this gap as both PND and breastfeeding may be affected by COVID‐19, and have important long‐term effects on women's and infant's health. A cross‐sectional internet‐based survey was conducted with 3253 post‐natal women from five countries: Brazil, South Korea, Taiwan, Thailand, and the United Kingdom from July to November 2021. The results showed that women who intended to breastfeed during pregnancy had lower odds of having PND than women who did not intend to. Women who had no breastfeeding intention but actually breastfed had greater odds (AOR 1.75) of having PND than women who intended to breastfeed and actually breastfed. While there was no statistical significance in expressed breast milk feeding in multivariable logistic regression models, women who had shorter duration of breastfeeding directly on breast than they planned had greater odds (AOR 1.58) of having PND than those who breastfed longer than they planned even after adjusting for covariates including COVID‐19‐related variables. These findings suggested the importance of working with women on their breastfeeding intention. Tailored support is required to ensure women's breastfeeding needs are met and at the same time care for maternal mental health during and beyond the pandemic.

## Key messages

- This study identified independent associations of breastfeeding intention and actual breastfeeding practices with PND after adjustment for COVID‐19‐related covariates.
- Although women who intended to breastfeed during pregnancy were less likely to have PND, those who did not intend to breastfeed but did breastfeed were more likely to have PND than those who intended to breastfeed and breastfed.
- Women with shorter (vs. longer) than planned duration of breastfeeding on breast were more likely to have PND.
- Health care providers and policymakers should ensure tailored support is available for women's infant feeding plans and mental health.

## 1 INTRODUCTION

Post‐natal depression (PND) is a depressive disorder that commences within 4 weeks following childbirth based on the Diagnostic and Statistical Manual of Mental Disorders Version 5 (DSM‐5; American Psychiatric Association, 2013) or 6 weeks following childbirth based on the International Classification of Diseases Version 10 (ICD‐10; World Health Organization [WHO], 2014). Women with PND may continue to suffer from depression 1 year after childbirth or longer (Slomian et al., 2019). PND not only negatively affects the woman's psychological health and quality of life but also her infant's physical, cognitive, social and behavioural development (Slomian et al., 2019). A recent systematic review including 565 pre‐COVID‐19 pandemic studies from 80 countries reported an overall PND prevalence of 17.22% (Wang et al., 2021), with variations across countries ranging from 6.48% in Denmark to 60.93% in Afghanistan, while 20.51% in Brazil, 22.50% in Korea, 21.65% in Taiwan, 12.52% in Thailand, and 21.50% in the United Kingdom (UK; Wang et al., 2021). A higher rate of PND may be observed in some countries due to possible factors, such as food insecurity (Ezzeddin et al., 2018), unemployment (Lewis et al., 2017), unplanned pregnancy (Barton et al., 2017), income inequality, maternal mortality, and infant mortality (Hahn‐Holbrook et al., 2018).

With the advent of the COVID‐19 pandemic, infection preventative measures such as self‐isolation, social distancing, facemask wearing and restricted hospital visits from partners or relatives have been put in place in hospitals and ‘hotspot’ areas to control the transmission of the virus in many countries. Studies undertaken during the first year of COVID‐19 pandemic have reported high rates of PND. For example, a survey conducted with 614 post‐natal women between April 2020 and May 2020 in the UK found that 43% of women had an Edinburgh post‐natal Depression Scale (EPDS) score ≥13, indicating a major post‐natal depressive disorder (Fallon et al., 2021). About 35% of 162 post‐natal women in London between May 2020 and June 2020 were assessed to have EPDS ≥ 13 (Myers & Emmott, 2021). A survey of 184 post‐natal women from two hospitals in Brazil between 8th June 2020 and 23rd December 2020 reported a 38.8% PND rate (EPDS ≥ 12; Galletta et al., 2022). An Italian study presented that 23.03% of 152 post‐natal women in a hospital who filled in the EPDS questionnaire on the second post‐natal day at hospital discharge between 22nd February 2020 and 18th May 2020 had an EPDS score ≥ 12 compared to 11.56% of 147 women from the nonconcurrent control group in 2019 (*p* < 0.001; Zanardo et al., 2021). These findings are concerning due to the negative and long‐term impact of PND on the woman, her infants and her family (Myers & Johns, 2018; Slomian et al., 2019; Tammentie et al., 2004).

The short‐ and long‐term benefits of breastfeeding to the health of women and infants have been well documented (Victora et al., 2016). Breastfeeding has also been found to provide protection against infectious respiratory diseases such as severe acute respiratory syndrome coronavirus 2 (SARS‐CoV‐2) which caused COVID‐19 (Didikoglu et al., 2021; Verd et al., 2021). For women diagnosed or suspected of COVID‐19, breastfeeding remains the recommended infant feeding practice but with precautions, such as wearing a facemask during feeding and good hand hygiene (WHO, 2020). A narrative review that included 12 studies between January 2020 and January 2021 reported that COVID‐19 had both positive and negative impacts on women's breastfeeding plans (Pacheco et al., 2021). The positive impact included that some women were able to spend more time at home with their infants and increased breastfeeding duration. The negative impact included reduced breastfeeding duration and frequency, and earlier breastfeeding cessation due to increased childcare responsibilities at home and perceived lack of support from family and professionals (Pacheco et al., 2021).

Breastfeeding could be a protective factor for PND and reduce post‐natal depressive symptoms (Figueiredo et al., 2014). However, unmet breastfeeding expectations may increase the risk of PND in some women (Gregory et al., 2015). Borra et al. (2015) reported that among women who were not depressed before childbirth, those who had intended to breastfeed and actually breastfed had the lowest risk of PND, while those who had intended to breastfeed but did not breastfeed had the highest risk of PND. On the other hand, PND has been indicative of early breastfeeding cessation (Brown et al., 2016; Dias & Figueiredo, 2015).

Limited studies conducted during the COVID‐19 pandemic have investigated the relationships between PND and breastfeeding. Zanardo et al. (2021) study during the COVID‐19 lockdown in an Italian hospital showed that women who breastfed their infants exclusively at hospital discharge on the second post‐natal day had significantly lower EPDS scores compared to women who practised formula feeding and complementary feeding. In a study amongst Malaysian post‐natal women with premature infants at the beginning of the pandemic, Yahya et al. (2021) found women who were of a high risk of PND had less positive attitudes towards breastfeeding compared to those who had a low risk of PND. However, none of the studies during COVID‐19 explored the relationships between (un)changed breastfeeding plans and PND, and on an international level. Our study is the first that addresses this gap.

The aim of this article was to report the associations between breastfeeding intention, breastfeeding practices, and PND considering COVID‐19‐related factors among post‐natal women in five countries. This article formed part of a larger multi‐country project examining various aspects (including infant feeding, PND, social support, maternity care, COVID‐19 infection and COVID‐19 vaccination acceptance) of post‐natal women's experiences of having a baby during the COVID‐19 pandemic.

## 2 METHODS

### 2.1 Study design and participants

A cross‐sectional online survey was conducted in five countries: Brazil, South Korea, Taiwan, Thailand, and the UK from July 2021 to November 2021. The choice of countries was a convenience sample of countries that had similar PND rates pre‐COVID (around 21%), except Thailand (12.52%; Wang et al., 2021). A convenience sampling technique was used to recruit participants. Survey participants’ inclusion criteria were post‐natal women who were: (a) up to 6 months postpartum (b) living in one of the participating countries, (c) aged 18–49 years old (except in Taiwan 20–49 years old), and (d) literate in the residential country's official language. The recruitment information and the survey web link were advertised online and/or through hard copies of posters or flyers, with a quick response (QR) code where used, as planned by the study lead of each country. The researchers from each country distributed the survey information in the official language of the country (i.e., Portuguese, South Korean, Mandarin with traditional Chinese characters, Thai, and English) via various channels such as emails, social media (e.g., Twitter, Facebook, WhatsApp groups, Line groups, etc.), parenting online forums, personal networks, relevant health care services, and not‐for‐profit organisations, including services supporting breastfeeding, women, children and families.

### 2.2 Data collection

Data were collected anonymously via an online Google Form in each country's official language. Except for the outcome variable mentioned below, the survey questions were initially developed in English and subsequently translated into the official language of the participating country. Back translations were also undertaken. Online informed consent was obtained from all participants before they started the survey. All data were anonymised. Ethical approval was granted from each country's relevant ethical approval body (detailed in Section 2.4).

#### 2.2.1 Outcome variable

The outcome variable in this study is depressive symptoms. The EPDS, a 10‐item self‐report scale, was used to assess post‐natal women's mental health in the last 7 days, as stated in the EPDS, using Likert scales (scoring 0–3, with a total score ranging between 0 and 30; Cox et al., 1987; Khalifa et al., 2016). An EPDS cutoff point of 13 or above was used to classify those with depression. Validated EPDS versions in each country's official language were used.

#### 2.2.2 Independent variables

The independent variables were: (1) intention to breastfeed during pregnancy, (2) breastfeeding intention during pregnancy and actual breastfeeding practices during postpartum, (3) impact of COVID‐19 on baby fed directly from breast, and (4) Impact of COVID‐19 on feeding expressed breast milk.

1. Intention to breastfeed during pregnancy was asked with response option (‘yes’ or ‘no’ or ‘don't know’) and was categorised into (‘yes’ or ‘no/don't know’) in the analyses.
2. ‘Breastfeeding intention during pregnancy and actual breastfeeding practices during postpartum’ were analysed by combining two variables (A) breastfeeding intention during pregnancy and (B) breastfeeding practices at the time of survey completion to form the categories of (a) no intention and no breastfeeding, (b) no intention and ever breastfeeding, (c) intention and no breastfeeding, (d) intention and ever breastfeeding. The breastfeeding practice at the time of survey completion was asked with options (i) completed stop breastfeeding/expressing breast milk, (ii) still breastfeeding/expressing breast milk, and (iii) never initiated breastfeeding/expressing breast milk. (i) and (ii) were categorised as ‘ever breastfeeding’.
3. Impact of COVID‐19 on baby fed directly on breast was asked using the question ‘Does COVID‐19 affect your infant feeding behaviour?’ with response options (i) not intend to feed, (ii) shorter than intended, (iii) the same duration as intended, and (iv) longer than intended.
4. Impact of COVID‐19 on baby fed expressed breast milk was asked using the question ‘Does COVID‐19 affect your infant feeding behaviour?’ with response options (i) not intend to feed, (ii) shorter than intended, (iii) the same duration as intended, and (iv) longer than intended.

#### 2.2.3 Covariates

##### Socio‐demographic, obstetric, health and support characteristics

Socio‐demographic variables of women included country, mother's age, education level, work status, education level, residence and marital status. Obstetric variables were pregnancy intention, mode of childbirth, parity, birthweight and preterm birth. Health and support variables were (a) health problem of mother during pregnancy, delivery or postpartum, (b) infant feeding practices in the last 24 h of survey completion, (c) number of post‐natal care received with the question ‘How many times have you received post‐natal care by health personnel within the 42 days (6 weeks) of childbirth?’, and (d) social support post‐birth (scores)measured using a six‐item self‐report scale—Maternity Social Support Scale (MSSS; Webster et al., 2000).

##### COVID‐19 knowledge, attitudes and practices (KAP), and beliefs of breastfeeding in relation to COVID‐19

Questions regarding KAP on infection prevention and control measures against COVID‐19 were drawn and modified from previous studies (Hussain et al., 2020; Islam et al., 2020). Nine questions were included to assess knowledge of COVID‐19 by answering if a statement (e.g., ‘COVID‐19 can NOT spread through respiratory droplets of infected individuals’) was ‘true’, ‘false’ or ‘do not know’. A correct answer was awarded a score indicating ‘adequate answer’. A total score ranged from 0 to 9, with a higher score indicating better knowledge. Attitudes toward the severity and prevention of COVID‐19 had seven questions (e.g., ‘Social distancing is important to prevent COVID‐19’) with 5‐point Likert scales (‘Strongly disagree’, ‘disagree’, ‘undecided’, ‘agree’ and ‘strongly agree’. A total score ranged from 7 to 35. A higher score indicated a more positive attitude. Precaution practices of COVID‐19 contain six questions including questions such as ‘During the last 7 days, did you avoid touching eyes, nose and mouth with unwashed hand?’ with 4‐point Likert scales (‘never’, ‘occasionally’, ‘sometimes’ and ‘always’). A total score ranged from 6 to 24, with higher scores indicating a more adequate practice.

Six questions (e.g., If the mother is confirmed or suspected to have COVID‐19, the mother should not breastfeed) were developed for breastfeeding beliefs in relation to infection prevention and control measures for COVID‐19 based on WHO's (2020) breastfeeding Q&A. The answer options were ‘disagree’, ‘uncertain’ and ‘agree’. A total score ranges from 0 to 12, with higher score showing a more positive breastfeeding belief.

##### COVID‐19 impact variables

Impact of COVID‐19 on food security was assessed before and during COVID‐19 using two questions: ‘Did you ever run out of food before the end of the month or cut down on the amount you ate to feed others in 2019 BEFORE COVID‐19?’ and ‘Did you ever run out of food before the end of the month or cut down on the amount you ate to feed others DURING COVID‐19 in 2020‐2021?’ The two variables were combined and categorised into (i) no change: insecure to insecure, (ii) worse: secure to insecure, (iii) better: insecure to secure and (iv) no change: secure to secure. Variables regarding COVID‐19 positive diagnosis (yes or no), and COVID‐19 vaccination uptake (yes or no) were also included.

### 2.3 Statistical analysis

The data analyses were conducted using SAS statistical software package version 9.3 (SAS Institute Inc.). Descriptive statistics were used to analyse categorical variables (frequencies and percentages) and continuous variables (mean and standard deviation [SD]). To test the association between PND (outcome variable) and the four independent variables: (1) intention to breastfeeding during pregnancy, (2) breastfeeding intention during pregnancy and actual breastfeeding practices, 3) impact of COVID‐19 on baby fed directly from breast, and (4) Impact of COVID‐19 on feeding expressed breast milk, bivariate associations were assessed using chi‐square test and simple logistic regression. Subsequently, to assess the impact of each independent variable on PND, adjusted for other variables, three multivariable logistic regression models were used: (i) model I adjusted for socio‐demographic, obstetric, health and support characteristics; (ii) model II adjusted for the covariates in model I and COVID‐19 related KAP and belief towards breastfeeding during COVID‐19; and (iii) model III adjusted for the covariates in model II and COVID‐19 impact variables: food security status before and during COVID‐19, positive COVID‐19 diagnose, COVID‐19 vaccination uptake. Adjusted odds ratio (AOR) with a 95% confidence interval (CI) was used to assess the strength of these associations.

### 2.4 Ethical statement

Online informed consent was obtained from all participants before they started the survey. All data were anonymised. Ethical approval was granted from each country's relevant ethical approval body: Brazil (Ethical Committee of Universidade Federal de São Paulo, reference number: 4.858.900), Taiwan (Institutional Review Board of the National Yang Ming Chiao Tung University, YM110060E), Thailand (a Mahidol University Ethical Committee, 2021/03‐042), South Korea (Institutional Review Board of Catholic Kkottong University, 2‐7008080‐A‐N‐01‐202103‐HR‐003), and the UK (Psychiatry Nursing and Midwifery Research Ethics Subcommittee at King's College London, HR/DP‐20/21‐22651, RESCM‐20/21‐22651).

## 3 RESULTS

A total of 3253 eligible responses were received from post‐natal women living in five countries: Brazil, South Korea, Taiwan, Thailand and the UK. Table 1 shows the characteristics of the participants. The majority of all women were between 30 and 39 years old (61.6%), had a university or higher degree (75.8%), lived in urban areas (72.6%), were married (95.5%), were on either paid or unpaid maternity leave (59.3%), had vaginal birth (61.0%), were primiparous (57.0%), and had received at least one dose of COVID‐19 vaccine (72.2%). About 74% (73.5%) of all women fed their baby directly on breast, 40.6% fed their baby with infant formula, and 38.3% with expressed breast milk in the past 24 h of survey completion (Table 1).

**Table 1:** Characteristics of study participants

	All	Brazil	Taiwan	Thailand	South Korea	UK	*p*
	*N* = 3252 (100%)	*N* = 560 (17.2%)	*N* = 614 (18.9%)	*N* = 840 (25.8%)	*N* = 381 (11.7%)	*N* = 858 (26.4%)	
	*n* (%)	*n* (%)	*n* (%)	*n* (%)	*n* (%)	*n* (%)	
Maternal age							<0.0001
18–29	1094 (33.6)	164 (29.3)	204 (33.2)	489 (58.2)	51 (13.4)	186 (21.7)	
30–39	2005 (61.6)	360 (64.3)	397 (64.6)	318 (37.9)	311 (81.6)	619 (72.1)	
>/=40	154 (4.7)	36 (6.4)	13 (2.1)	33 (3.93)	19 (5.0)	53 (6.2)	
Work status							<0.0001
Yes	564 (17.3)	87 (15.6)	28 (4.6)	357 (42.5)	61 (16.0)	31 (3.6)	
No	762 (23.4)	104 (18.6)	99 (16.1)	312 (37.1)	197 (51.7)	50 (5.8)	
On paid maternity leave	1583 (48.7)	335 (60.0)	312 (50.8)	121 (14.4)	84 (22.1)	731 (85.2)	
On unpaid maternity leave	343 (10.6)	33 (5.9)	175 (28.5)	50 (5.95)	39 (10.2)	46 (5.4)	
Educational level							<0.0001
Secondary school or lower	787 (24.2)	85 (15.2)	35 (5.7)	458 (54.5)	34 (8.9)	175 (20.4)	
University or higher	2465 (75.8)	475 (84.8)	579 (94.3)	382 (45.5)	347 (91.1)	682 (79.6)	
Residence							<0.0001
Urban	2360 (72.6)	542 (97.1)	535 (87.1)	382 (45.5)	360 (94.5)	541 (63.0)	
Rural	891 (27.4)	16 (2.9)	79 (12.9)	458 (54.5)	21 (5.5)	317 (37.0)	
Martial status							<0.0001
Married	3105 (95.5)	521 (93.0)	605 (98.5)	759 (90.4)	380 (99.7)	840 (97.9)	
Others	148 (4.6)	39 (7.0)	9 (1.4)	81 (9.6)	1 (0.3)	18 (2.1)	
Intended pregnancy							<0.0001
Yes	2614 (80.4)	465 (83.0)	414 (67.4)	705 (83.9)	268 (70.3)	762 (88.8)	
No	639 (19.6)	95 (17.0)	200 (32.6)	135 (16.1)	113 (29.7)	96 (11.2)	
Parity							<0.0001
1	1853 (57.0)	393 (70.2)	424 (69.1)	436 (51.9)	207 (54.6)	393 (45.8)	
>1	1398 (43.0)	167 (29.8)	190 (30.9)	404 (48.1)	172 (45.4)	465 (54.2)	
Mode of childbirth							<0.0001
Vaginal	1985 (61.0)	272 (48.6)	417 (67.9)	489 (58.2)	251 (65.9)	556 (64.8)	
Caesarean	1268 (39.0)	288 (51.4)	197 (32.1)	351 (41.8)	130 (34.1)	302 (35.2)	
Birthweight							0.0045
<2.5 kg	250 (7.7)	43 (7.7)	57 (9.3)	81 (9.6)	18 (4.7)	51 (5.9)	
2.5–3.5 kg	3002 (92.3)	516 (92.3)	557 (90.7)	759 (90.4)	363 (95.3)	807 (94.1)	
>3.5 kg	837 (25.7)	137 (24.5)	63 (10.3)	101 (12.0)	79 (20.7)	457 (53.3)	
Preterm birth	399 (12.3)	52 (9.3)	60 (9.8)	173 (20.6)	48 (12.6)	66 (7.7)	<0.0001
Health problems of mother							<0.0001
Yes	1422 (43.7)	205 (36.6)	450 (73.3)	196 (23.3)	80 (21.0)	491 (57.2)	
No	1831 (56.3)	355 (63.4)	164 (26.7)	644 (76.7)	301 (79.0)	367 (42.8)	
Number of post‐natal care received							<0.0001
Never	421 (12.9)	49 (8.7)	9 (1.5)	246 (29.3)	90 (23.6)	27 (3.2)	
1–2	1194 (36.7)	315 (56.2)	55 (8.9)	486 (57.9)	199 (52.2)	139 (16.2)	
3	363 (11.16)	78 (13.93)	43 (7)	43 (5.12)	36 (9.45)	163 (19)	
4	1275 (39.19)	118 (21.07)	507 (82.57)	65 (7.74)	56 (14.7)	529 (61.66)	
Social support (mean, SD)	25.16 (3.7)	24.67 (3.6)	25.29 (3.4)	24.95 (3.7)	23.53 (4.2)	26.32 (3.1)	<0.0001
Ever diagnosed as COVID‐19 positive (yes)	417 (12.8)	140 (25.0)	1 (0.2)	143 (17.0)	6 (1.6)	127 (14.8)	<0.0001
Ever received COVID‐19 vaccine (yes)	2348 (72.2)	543 (97.0)	484 (78.8)	469 (55.8)	96 (25.2)	756 (88.1)	<0.0001
Impact of COVID‐19 on food insecurity							<0.0001
Insecure to insecure	298 (9.2)	19 (3.4)	–	236 (28.1)	21 (5.5)	22 (2.6)	
Worse	340 (10.5)	75 (13.5)	–	180 (21.4)	13 (3.4)	72 (8.4)	
Better	27 (0.8)	5 (0.9)	–	13 (1.6)	5 (1.3)	4 (0.5)	
Secure to secure	2584 (79.5)	457 (82.2)	614 (100.0)	411 (48.9)	342 (89.8)	760 (88.6)	
Breastfeeding in the last 24 h							
Directly on breast	2392 (73.5)	508 (90.7)	333 (54.2)	544 (64.8)	274 (71.9)	733 (85.4)	<0.0001
Expressed breast milk	1246 (38.3)	77 (13.8)	323 (52.6)	503 (59.9)	192 (50.4)	151 (17.6)	<0.0001
Infant formula	1321 (40.6)	111 (19.8)	450 (73.3)	328 (39.1)	219 (57.5)	213 (24.8)	<0.0001
Solid, semi‐solid or soft foods	388 (11.9)	39 (7.0)	133 (21.7)	66 (7.86)	60 (15.8)	90 (10.5)	<0.0001

Table 1 shows study participants’ characteristics were statistically significantly different by country. Brazil (25.0%) had the highest percentage of women who had ever been diagnosed as COVID‐19 positive while the lowest was in Taiwan (0.2%). The highest percentage of women who had received at least one dose of COVID‐19 vaccine was in Brazil, followed by the UK (88.1%), Taiwan (78.8%), Thailand (55.8%), and South Korea (25.2%). The rate of PND in all women was 29.3%, ranging from 37% in South Korea, 32.4% in Thailand, 30.4% in Brazil, 26.6% in the UK, to 23.3% in Taiwan (Table 2).

**Table 2:** Bivariate associations between breastfeeding intention, breastfeeding practices and post‐natal depression

		Pooled			Brazil			Taiwan		
		PND (EPDS score ≥ 13)			PND (EPDS score ≥ 13)			PND (EPDS score ≥ 13)		
	Total	Yes	No		Yes	No		Yes	No	
	3252 (100)	954 (29.3)	2299 (70.7)	*p*	170 (30.4)	390 (69.6)	*p*	143 (23.3)	471 (76.7)	*p*
**Breastfeeding intention**										
Intention to breastfeeding during pregnancy (yes)	2931 (90.1)	825 (86.5)	2106 (91.6)	<0.0001	166 (97.7)	388 (99.5)	0.0518	114 (79.7)	392 (83.2)	0.3347
**Breastfeeding intention and breastfeeding practices**										
No intention and no breastfeeding	56 (1.7)	17 (1.8)	39 (1.7)	<0.0001	1 (0.6)	0 (0.0)	0.0903	0 (0.0)	7 (1.5)	0.2364
No intention and ever breastfeeding	266 (8.2)	112 (11.8)	154 (6.7)		3 (1.8)	2 (0.5)		29 (20.3)	72 (15.3)	
Intention and no breastfeeding	70 (2.2)	22 (2.3)	48 (2.1)		1 (0.6)	8 (2.1)		0 (0.0)	2 (0.4)	
Intention and ever breastfeeding	2853 (87.9)	802 (84.2)	2051 (89.5)		164 (97.0)	37.3 (97.4)		114 (79.7)	390 (82.8)	
**Impact of COVID‐19 on baby fed directly on breast**										
Not intend to feed	489 (15.1)	126 (13.2)	363 (15.8)	<0.0001	24 (14.1)	56 (14.5)	0.8798	10 (7.0)	63 (13.4)	0.009
Shorter than I intended	603 (18.6)	237 (24.8)	366 (16.0)		21 (12.4)	39 (10.1)		60 (42.0)	137 (29.1)	
The same duration as I intended	1746 (53.7)	464 (48.6)	1282 (55.9)		105 (61.8)	247 (64.0)		51 (35.7)	226 (48.0)	
Longer than I intended	411 (12.7)	127 (13.3)	284 (12.4)		20 (11.8)	44 (11.4)		22 (15.4)	45 (9.6)	
**Impact of COVID‐19 on expressed breast milk**										
Not intend to feed	895 (27.6)	240 (25.2)	655 (28.5)	<0.0001	57 (33.5)	149 (38.6)	0.0189	12 (8.4)	39 (8.3)	0.0315
Shorter than I intended	654 (20.1)	249 (26.1)	405 (17.7)		40 (23.5)	55 (14.3)		54 (37.8)	138 (29.3)	
The same duration as I intended	1325 (40.8)	354 (37.1)	971 (42.3)		65 (38.2)	146 (37.8)		51 (35.7)	232 (49.3)	
Longer than I intended	375 (11.5)	111 (11.6)	264 (11.5)		8 (4.7)	36 (9.3)		26 (18.2)	62 (13.2)	

	Thailand			South Korea			UK		
	PND (EPDS score ≥ 13)			PND (EPDS score ≥ 13)			PND (EPDS score ≥ 13)		
	Yes	No		Yes	No		Yes	No	
	272 (32.4)	568 (67.6)	*p*	141 (37.0)	240 (63.0)	*p*	228 (26.6)	630 (73.4)	*p*
**Breastfeeding intention**									
Intention to breastfeeding during pregnancy (yes)	223 (82.0)	535 (94.2)	<0.0001	102 (72.3)	182 (75.8)	0.4499	220 (96.5)	609 (96.7)	0.0900
**Breastfeeding intention and breastfeeding practices**									
No intention and no breastfeeding	10 (3.7)	11 (1.9)	<0.0001	3 (2.1)	8 (3.3)	0.6171	3 (1.3)	13 (2.1)	0.3726
No intention and ever breastfeeding	39 (14.3)	22 (3.9)		36 (25.5)	50 (20.8)		5 (2.2)	8 (1.3)	
Intention and no breastfeeding	16 (5.9)	32 (5.6)		1 (0.7)	1 (0.4)		4 (1.8)	5 (0.8)	
Intention and ever breastfeeding	207 (76.1)	503 (88.6)		101 (71.6)	181 (75.4)		216 (94.7)	604 (95.9)	
**Impact of COVID‐19 on baby fed directly on breast**									
Not intend to feed	72 (26.5)	193 (34.0)	<0.0001	9 (6.4)	20 (8.3)	0.6778	11 (4.8)	31 (4.9)	0.0016
Shorter than I intended	85 (31.3)	81 (14.3)		33 (23.4)	61 (25.4)		38 (16.7)	48 (7.6)	
The same duration as I intended	72 (36.5)	179 (31.5)		70 (49.7)	120 (50.0)		166 (72.8)	510 (81.0)	
Longer than I intended	43 (15.8)	115 (20.3)		29 (20.6)	39 (16.3)		13 (5.7)	41 (6.5)	
**Impact of COVID‐19 on expressed breast milk**									
Not intend to feed	73 (26.8)	162 (28.5)	0.0024	20 (14.2)	47 (19.6)	0.2120	78 (34.2)	258 (41.0)	0.0005
Shorter than I intended	79 (29.0)	101 (17.8)		37 (26.2)	64 (26.7)		39 (17.1)	47 (7.5)	
The same duration as I intended	80 (29.4)	201 (35.4)		60 (42.6)	104 (43.3)		98 (43.0)	288 (45.7)	
Longer than I intended	40 (14.7)	104 (18.3)		24 (17.0)	25 (10.4)		13 (5.7)	37 (5.9)	

Table 2 demonstrates the percentages of four independent variables related to breastfeeding intention and/or breastfeeding practices and their bivariate associations with PND. About 90% of all women intended to breastfeed during pregnancy. The pooled data from all countries showed that there was a significant association between breastfeeding intention during pregnancy and PND (*p* < 0.0001), although a statistical significance was only shown in Thailand (*p* < 0.0001).

Regarding women's breastfeeding intention during pregnancy and actual breastfeeding practice during postpartum, 87.9% intended to breastfeed during pregnancy and actually breastfed, while 8.2% did not intend to and actually breastfed, 2.2% intended but did not breastfeed and 1.7% did not intend and did not breastfeed. The chi‐square test revealed that there was a significant association with PND in the pooled data (*p* < 0.0001). At the country level, a statistical significance was only seen in Thailand (*p* < 0.0001).

Women were asked ‘Does COVID‐19 affect your infant feeding behaviour?’ with two types of breastfeeding: directly on breast and expressed breast milk. About 54% (53.7%) of all women responded to breastfeeding directly on breast for the same duration as planned, while 18.6% shorter than planned, and 12.7% longer than planned. The bivariate analysis showed a significant association between the impact of COVID‐19 on breastfeeding directly on breast and PND in the pooled model (*p* < 0.001) and by country in Taiwan, Thailand and the UK (*p* < 0.01). In terms of expressed breast milk feeding, 40.8% fed their baby expressed breast milk the same duration as planned, 20.1% shorter than planned and 11.5% longer than planned. The bivariate analysis revealed a significant association with PND in the pooled model (*p* < 0.0001) and in almost all countries (*p* < 0.05), except South Korea.

Table 3 shows the bivariate and multivariable associations between the four independent variables and PND covariates. In terms of breastfeeding intention during pregnancy, women who intended to breastfeed during pregnancy had lower odds of having PND than women who did not intend to (COR 0.59, 95% CI 0.46–0.74). After adjustment for covariates, the association attenuated but remained significant (AOR 0.66, 95% CI 0.50–0.87 in model 1, 0.66, 0.50–0.88 in model 2 and 0.65, 0.49–0.86 in model 3).

**Table 3:** Bivariate and multivariable associations between breastfeeding intention, breastfeeding practices and post‐natal depression

	Pooled		Model 1		Model 2		Model 3	
	*COR*	*(95% CI)*	*AOR*	*(95% CI)*	*AOR*	*(95% CI)*	*AOR*	*(95% CI)*
Intention to breastfeeding during pregnancy (yes)	0.59	(0.46–0.74)	0.66	(0.50–0.87)	0.66	(0.50–0.88)	0.65	(0.49–0.86)
**Breastfeeding intention and breastfeeding practices**								
No intention and no breastfeeding	1.12	(0.63–1.98)	0.74	(0.38–1.42)	0.72	(0.37–1.39)	0.73	(0.37–1.41)
No intention and ever breastfeeding	1.86	(1.44–2.41)	1.73	(1.28–2.34)	1.72	(1.27–2.32)	1.75	(1.29–2.36)
Intention and no breastfeeding	1.17	(0.70–1.95)	0.78	(0.43–1.42)	0.78	(0.43–1.42)	0.78	(0.43–1.42)
Intention and ever breastfeeding	1.00		1.00	–	1.00	–	1.00	–
**Impact of COVID‐19 on baby fed directly on breast**								
Not intend to feed	0.78	(0.58–1.04)	0.74	(0.53–1.03)	0.76	(0.54–1.06)	0.75	(0.54–1.05)
Shorter than intended	1.45	(1.11–1.89)	1.55	(1.13–2.14)	1.59	(1.15–2.19)	1.58	(1.15–2.18)
The same duration as intended	0.81	(0.64–1.02)	0.94	(0.72–1.22)	0.95	(0.73–1.24)	0.96	(0.74–1.26)
Longer than intended	1.00		1.00	–	1.00	–	1.00	–
**Impact of COVID‐19 on expressed breast milk**								
Not intend to feed	0.87	(0.67–1.14)	0.75	(0.55–1.02)	0.75	(0.55–1.02)	0.73	(0.54–1.00)
Shorter than intended	1.46	(1.11–1.92)	1.19	(0.87–1.62)	1.21	(0.89–1.65)	1.20	(0.88–1.64)
The same duration as intended	0.87	(0.67–1.12)	0.86	(0.65–1.14)	0.88	(0.66–1.16)	0.89	(0.67–1.17)
Longer than intended	1.00	–	1.00	–	1.00	–	1.00	–

*Note*: Model: 1 included covariates of country (Brazil, South Korea, Taiwan, Thailand or the UK), maternal age (18–29, 30–39 or 40–49), intended pregnancy (yes or no), mode of childbirth (vaginal or caesarean section), health problem of mother during pregnancy, delivery or postpartum (yes or no), work status (employed, on paid maternity leave, on unpaid maternity leave or housewife/unemployed), residence (urban or rural), marital status (married or others), parity (1 or 2+), birthweight (<2.5, 2.5–3.5 or >3.5 kg), preterm birth (yes or no), breastfeeding directly on breast in the last 24 h (yes or no), expressed breast milk in the last 24 h (yes or no), infant formula in the last 24 h (yes or no), solid, semi‐solid or soft foods in the last 24 h (yes or no), social support (score), number of post‐natal care (never, 1–2, 3 or 4+)

Model 2: covariates in Model 1+ COVID‐19 knowledge (score), attitudes (score), and practices (score) and breastfeeding belief (score)

Model 3: covariates in Model 2+ changes in food insecurity before and during covid‐19 (no change (insecure), worse, better, or no change (secure)), ever diagnosed as COVID‐19 positive (yes or no), COVID‐19 vaccination (yes or no)

Regarding women's breastfeeding intention during pregnancy and actual breastfeeding practice, women who did not intend to breastfeed but actually breastfed had greater odds (COR 1.86, 95% CI 1.44–2.41) of having PND than women who intended to breastfeed and actually breastfed in the bivariate model. After adjustment for covariates, the association remained significant, despite a weaker association (AOR 1.73, 95% CI 1.28–2.34 in model 1, 1.72, 95% CI 1.27–2.32 in model 2 and 1.75, 1.29–2.36 in model 3).

In respect of impact of COVID‐19 on breastfeeding, women who had shorter duration of either breastfeeding directly on breast or expressed breast milk than they intended to had greater odds (COR 1.45 and 1.46, respectively) of having PND than those who breastfed longer than they intended to in the bivariate model. In the multivariate models, while there was no significant association with expressed breast milk feeding, the association with breastfeeding directly on breast remained significant after adjustment for sociodemographic, obstetric, health and support characteristics (AOR 1.55, 95% CI 1.13–2.14 in model 1). After further adjustment for COVID‐19 KAP and belief towards breastfeeding in relation to COVID‐19 in model 2 and COVID‐19 impact variables in model 3, the associations became a bit greater (1.59, 1.15–2.19 and 1.58, 1.15–2.18, respectively).

## 4 DISCUSSION

To the best of our knowledge, this is the first international study investigating the associations between breastfeeding intention, breastfeeding practices and PND during the COVID‐19 pandemic. We focus the discussion on the associations of the outcome variable and independent variables stated in this article. Discussions specifically on (a) breastfeeding and (b) PND were presented in separate articles as separate topics (Chien et al., 2022; Coca et al., 2022). Some key pooled results from the five countries in this study were that those who intended to breastfeed during pregnancy had lower odds of having PND (*p* < 0.0001) while those who had no breastfeeding intention but actually breastfed (*p* < 0.0001), and ceased breastfeeding directly on breast earlier than planned had higher odds of having PND (*p* < 0.0001), and to cease breastfeeding (directly on breast and expressed breast milk) earlier than they planned (*p* < 0.0001) compared to those with no PND. Similar findings were shown in some pre‐pandemic findings. For example, Gregory et al. (2015) found that women who met their breastfeeding expectation had lower odds of post‐natal depressive symptoms compared to those who did not. However, our study did not establish causal relationships. For example, we do not know if women already had depressive symptoms before childbirth and then developed PND, which may affect their breastfeeding decisions; or women's decision to stop breastfeeding earlier than planned due to the impact of COVID‐19 may trigger their PND, although previous pre‐pandemic studies have reported bidirectional relationships between breastfeeding and PND (Dias & Figueiredo, 2015; Pope & Mazmanian, 2016). Nevertheless, our findings demonstrate the importance of supporting women to achieve their breastfeeding plans as well as when their infant feeding plans change while at the same time caring for their mental health.

We further analysed the association between the type of changes in breastfeeding plans and PND. We showed in pooled models statistically significant results before and after adjusting for confounders, including COVID‐19‐related factors, that women with no intention to breastfeed but actually breastfed had greater odds of having PND than women who intended to breastfeed and breastfed. This showed the importance of understanding women's breastfeeding intention, working with women on their breastfeeding intention, and their subsequent breastfeeding practices which may be different from their intention. Borra et al. (2015) reported a similar finding that among women who did not have depressive symptoms during pregnancy, breastfeeding increased the risk of PND in women who had not intended to breastfeed and decreased the risk of PND in women who had intended to breastfeed. Other statistically significant results in our pooled models before and after adjusting for confounders were that women who breastfed directly on breast for shorter duration than they planned had greater odds of having PND than those who breastfed longer than they planned. Many pre‐pandemic studies have shown that women who had PND were more likely to have a shorter breastfeeding duration than those who did not have PND (Butler et al., 2021; Dias & Figueiredo, 2015; Pope & Mazmanian, 2016). On the other hand, Costantini et al. (2021) conducted an online survey in the UK during the COVID‐19 lockdown with women whose children were aged 0–3 years old and found no statistically significant difference in post‐natal depressive scores as measured by Patient Health Questionnaire (PHQ‐9) between women who breastfed more than 6 months and those who breastfed less than 6 months. However, Costantini et al. (2021) did not investigate if women changed their breastfeeding plans.

As mentioned above, the statistically significant associations between the type of changes in breastfeeding plans and PND remained statistically significant after adjusting for confounders, including COVID‐19‐related factors. This indicated that COVID‐19‐related factors included in our analyses did not contribute hugely to the associations. However, COVID‐19's influence on women changing their breastfeeding plans was evident in some women's responses to the survey questions concerning: ‘Does COVID‐19 affect your infant feeding behaviour?’, with nearly a third of all women reporting changing their actual breastfeeding (directly on breast or expressed breast milk) duration to shorter or longer than planned. It is unclear in our study which specific aspects of COVID‐19 impacted on these women changing their breastfeeding duration which were associated with PND. An online survey of UK post‐natal women over 4 weeks in May 2020 and June 2020 found that women ceased breastfeeding due to COVID‐19‐related concerns, such as lockdown and lack of support (Brown & Shenker, 2021). Piankusol et al. (2021) survey conducted between 17th July 2020 and 17th October 2020 reported that lack of family support with infant feeding was the risk factor associated with changing breastfeeding practices (e.g., reduced breastfeeding frequency) among post‐natal women under COVID‐19 lockdown in Thailand. A narrative review including studies during COVID‐19 from January 2020 to January 2021 reported a preliminary finding that factors during the pandemic such as separation from their baby, experiencing breastfeeding challenges and poorer support from family and health care professionals influenced women experiencing negative breastfeeding experiences which led to negative psychological outcomes (e.g., sadness; Pacheco et al., 2021). It is worth noting that these empirical studies were conducted within about 1 year of the COVID‐19 pandemic. Our data collection took place between July 2021 and November 2021, which was more than 1 year into the COVID‐19 pandemic when women might be more adjusted to living in the COVID‐19 pandemic. Our participants became pregnant and gave birth during the pandemic, with more than 70% of all participants having at least one dose of the COVID‐19 vaccine. Furthermore, face‐to‐face breastfeeding support which was reduced or temperately halted at the first year of the pandemic may have increased in some geographical areas and countries at the second year of the pandemic, although not yet returned to the pre‐pandemic level (UNICEF, 2021). Future research employing qualitative research methods can be conducted to understand the specific aspects of COVID‐19 that impacted on women's breastfeeding plans and mental health outcomes, especially when ‘living with COVID’ becomes a norm.

The importance of receiving support from health care professionals, partners, family members, and friends for breastfeeding and PND has consistently been shown in studies before and during COVID‐19 (da Silva Tanganhito et al., 2020; Myers & Emmott, 2021; Pacheco et al., 2021). The statistically significant associations found in our study between breastfeeding intention, breastfeeding practices and PND further illustrate the importance of providing effective breastfeeding support from health care professionals, partners and families to tailor infant feeding support to women's infant feeding needs and decisions, and to minimise the risk of PND. A Canadian prospective pre‐COVID study reported that women who experienced breastfeeding challenges scored lower EPDS scores when they did not report a negative experience with breastfeeding support (Chaput et al., 2016). This highlighted the need of positive and high‐quality breastfeeding support for post‐natal women to reduce the risk of PND (Chaput et al., 2016). There are few interventions to support women at risk of PND with breastfeeding. Reach Out, Stand Strong, Essentials for new mothers (ROSE) study which utilised a group interpersonal therapy to promote social support and self‐care in low‐income pregnant women at risk of PND has indicated a positive outcome of increased breastfeeding duration among women receiving the therapy, although further evidence is needed (Kao et al., 2015). Future interventions to support breastfeeding among post‐natal women at risk of PND or with PND should be developed and evaluated. Importantly, how interventions can be delivered when ‘living with COVID’ with desired preventative measures should be considered in the design of interventions.

In a study analysing stakeholder perspectives on companionship for women in antenatal and intrapartum care, Thomson et al. (2022) concluded a high prevalence of inconsistent policies in UK maternity services during the pandemic, in which psychological impacts were overlooked to prevent the spread of infection. Moreover, recent COVID‐19 studies continued to show the impact of COVID‐19 on breastfeeding support. For example, a lack of breastfeeding support was reported from health care services in Brazil during the pandemic (Gonçalves‐Ferri et al., 2021). A UK survey of infant feeding specialists/workers in November 2021 reported reduced face‐to‐face support due to reduced staff capacity, new COVID regulations, challenges with finding suitable venues and staff redevelopment (UNICEF, 2021). Innovate solutions should be developed and implemented to deliver high‐quality and safe maternity services including breastfeeding and mental health support. However, it is worth noting the staff capacity and redeployment issues during COVID‐19 highlighted above (UNICEF, 2021). Women's partners and families may also experience mental health difficulties themselves (van den Heuvel et al., 2022) and need support to be able to support women (Chang et al., 2021). Breastfeeding peer supporters were found to be beneficial in not only providing practical support for breastfeeding but also emotional/psychological support, as well as decreasing social isolation (Chang et al, 2022), which was reported as a challenge for post‐natal women during the pandemic due to restrictive measures and lack of support (Ipsos MoRI, 2021). Some peer support, including face‐to‐face in‐person interactions, continued to be provided during the COVID‐19 lockdown (Hann et al., 2021). Evaluating and learning from these support services may help inform the development of improved breastfeeding and mental health support with integration into health services for women and their families.

### 4.1 Limitations

This study has several limitations. Due to the use of convenience sampling, generalisability may not be appropriate to all post‐natal women in the participating countries and other countries. Other limitations of this study include the nature of assessing breastfeeding and PND as we used self‐report assessments with retrospective data, which increase the risk of eliciting social desirability bias and may lead to recall bias. We used the EPDS scale with a cutoff point of 13, in which previous research has shown that identifying PND using a tool, such as EPDS, may be insufficient to recognise women who need support (Fellmeth et al., 2019). Further, we did not ask if women had antenatal depression or previous mental illness or received support for previous mental illness. Despite the limitations of this study, this study has certain strengths. Our study is the first that addresses the relationships between (un)changed breastfeeding plans and PND. Furthermore, being an international study across five countries on this important topic during COVID‐19 is a strength and the results can also inform practices and policies for future pandemics.

## 5 CONCLUSIONS

Our study highlighted that breastfeeding intention at pregnancy and change of breastfeeding plans were associated with PND during the COVID‐19 pandemic. Further investigation is needed to identify effective breastfeeding interventions in preventing PND and reducing post‐natal depressive symptoms, combining ‘living with COVID’ desired preventative measures. Working with women on their breastfeeding intention during pregnancy is important. post‐natal care should include supporting women's breastfeeding decision‐making and identify breastfeeding/infant feeding support needs for women more likely to be at risk of PND. It is also essential that policymakers and health care providers provide guidance and take actions on mitigating the long‐term effects of unmet breastfeeding plans and PND, and preventing/reducing occurrences of PND and post‐natal depressive symptoms and negative breastfeeding outcomes for women during the pandemic and beyond.

## AUTHOR CONTRIBUTIONS

Seo A. Hong and Yan‐Shing Chang initiated and developed the study. Yan‐Shing Chang, Kelly P. Coca, Li‐Yin Chien, Eun Y. Lee and Seo A. Hong led on data collection. Yan‐Shing Chang, Seo A. Hong and Kan M. C. Li interpreted the results. Yan‐Shing Chang and Kan M. C. Li drafted the manuscript. All authors have read and approved the final version of the manuscript for publication.

## CONFLICT OF INTEREST

The authors declare no conflict of interest.

## Data Availability

The data underlying this study cannot be made publicly available, since approval was not obtained from study participants to make their data openly available.
